# microRNA-124-3p attenuates myocardial injury in sepsis via modulating SP1/HDAC4/HIF-1α axis

**DOI:** 10.1038/s41420-021-00763-y

**Published:** 2022-01-28

**Authors:** Mei Wu, Zehong Huang, Wenfang Huang, Mengyu Lin, Weifeng Liu, Kexuan Liu, Cai Li

**Affiliations:** Department of Anesthesiology, Nanfang Hospital, Southern Medical University, Guangzhou, 510515 Guangdong China

**Keywords:** Cell biology, Diseases

## Abstract

Sepsis-induced cardiac dysfunction can lead to death in sepsis. In this case, we targeted to explore in detail the relative mechanism of microRNA (miR)-124-3p in sepsis-induced myocardial injury via the specific protein 1/histone deacetylase 4/hypoxia-inducing factor 1α (SP1/HDAC4/HIF-1α) axis. Septic rats were modeled by cecal ligation puncture while in vitro septic cardiomyocyte H9C2 were induced by lipopolysaccharide (LPS). miR-124-3p/SP1/HDAC4/HIF-1α expression levels in myocardial tissues of septic rats and LPS-treated H9C2 cells were measured. miR-124-3p overexpression and SP1 silencing assays were implemented on LPS-treated H9C2 cells to explore theirs actions in inflammation, oxidative stress and cell apoptosis. The interactions of miR-124-3p, SP1, and HDAC4 were testified. miR-124-3p was lowly expressed while SP1, HDAC4, and HIF-1α were highly expressed in sepsis. Upregulation of miR-124-3p ameliorated inflammation, oxidative stress, and apoptosis of LPS-treated H9C2 cells. Silencing SP1 improved LPS-induced damage to cardiomyocytes. miR-124-3p targeted SP1 and HDAC4 interacted with SP1. SP1 overexpression antagonized miR-124-3p upregulation-induced improvements in LPS-induced cardiomyocyte damage. This study illustrates that miR-124-3p improves myocardial injury in septic rats through targeted regulation of SP1 to mediate HDAC4/HIF-1α.

## Introduction

Sepsis is featured by inflammatory disorders, which is an inflammatory immune response stimulated by infection [1]. Patients with sepsis complain a variety of immunological changes that ultimately lead to immunosuppression [2]. In fact, sepsis-related mortality reduction depends on early diagnosis and prompt empirical antibiotic therapy [3]. About 40–60% of septic patients manifest myocardial dysfunction that is characterized by myocardial systolic and diastolic dysfunction [4]. Several methods have been developed to prevent sepsis-induced myocardial dysfunction, but effective treatments are still inaccessible. Facing to the challenge to treat myocardial injury induced by sepsis, much more studies shall be conducted to explore novel treatment options.

Belonging to microRNA (miRNA) family, aberrantly expressed miR-124 is connected with inflammation and increased disease risk of sepsis [5]. Besides, overexpression of miR-214 could attenuate apoptosis of cardiomyocytes in sepsis [6]. More importantly, miR-124-targeted therapy is useful to reduce the production of pro-inflammatory cytokines in sepsis [7]. Also, miR-124 has been testified to alleviate lung injury in mice with sepsis [8, 9]. Specific protein 1 (SP1) is reported to associate with lipopolysaccharide (LPS)-induced vascular hypocontractility and mortality [10]. Additionally, SP1 could activate zinc finger antisense 1, thereafter to accelerate the development of cardiac dysfunction induced by sepsis [11]. Histone deacetylases (HDAC) inhibitors are applied to treat sepsis [12]. Concretely, excessively expressed nuclear HDAC4 has been detected in cecal ligation and puncture (CLP) rats [13] and also overexpressed HDAC4 stimulates the progression of myocardial injury [14]. It is lately documented that HDAC4 elevation causes H9C2 cell death and mitochondrial dysfunction in hypoxia/reoxygenation injury [15]. Hypoxia-inducing factor 1 (HIF-1) signaling pathway is the channel that monocytes undergo reprogramming to generate immunosuppression in the late stage of sepsis [16]. It has been proved that downregulating the activated HIF-1α, the HDAC4 client transcription factor, could improve the prognosis of sepsis [17] and relieve myocardial injury during sepsis [18]. Enlightened by those studies, we would wonder that whether miR-124-3p could interact with SP1, thereby affecting HDAC4/HIF-1α axis to attenuate myocardial injury in sepsis.

## Results

### Sepsis induces severe myocardial injury and inflammation in rats

**Fig. 1:**
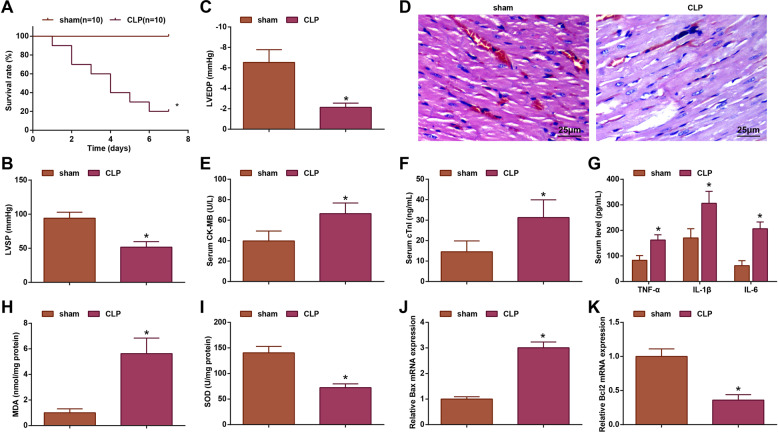
Sepsis induces myocardial injury and inflammation in rats. **A** The 7-day survival rate after CLP; **B** LVSP value in rats; **C** LVEDP value in rats; **D** HE staining detected pathological damage of rat myocardial tissues; **E** ELISA detected CK-MB level in rats; **F** ELISA detected cTn-I level in rats; **G** ELISA detected TNF-α, IL-1β and IL-6 levels in rat serum; **H** MDA level in rat myocardial tissues; **I** SOD activity in rat myocardial tissues; **J** RT-qPCR detected Bax mRNA expression in rats; **K** RT-qPCR detected Bcl-2 mRNA expression in rats; the measurement data were expressed as mean ± standard deviation, *n* = 6, *P < 0.05 compared with the sham group.

In septic rats, myocardial contraction and diastolic function were weakened, which often manifested as heart failure, impaired myocardial function, increased mortality, and increased inflammatory factors [19, 20]. CLP was adopted to animal modeling of sepsis. Observation of the survival rate of rats within 7 days showed that there was no death in the sham group, while the survival rate of rats in the CLP group was 20% (Fig. 1A). In the experiments, decreased LVSP value and increased LVEDP value were seen in septic rats (Fig. 1B, C), suggesting the impaired myocardial contraction and diastolic function. After HE staining, cardiomyocytes of sham-operated rats were normally constructed with tightly arranged fibromyoma filament bundles but without edema, congestion, degeneration and necrosis. Septic rats showed pathological changes such as local necrosis, interstitial edema, myocardial fiber rupture, and inflammatory cell infiltration (Fig. 1D).

After animal modeling, myocardial function-related factors (CK-MB and cTnI) of rats were tested and their levels in septic rats were both increased (Fig. 1E, F). Similarly, inflammatory factors (TNF-α, IL-1β and IL-6) were also found to increase in serum of septic rats (Fig. 1G).

MDA is an important parameter that reflects anti-oxidation potential, lipid peroxidation rate and intensity, and indirectly reflects the degree of tissue peroxidation damage. SOD could catalyze the disproportionation of superoxide anion radicals into hydrogen peroxide and oxygen, and the superoxide anion radicals produced are normal metabolites in organisms. SOD possesses therapeutic effect for MI [21, 22]. MDA was determined by thiobarbituric acid method while SOD activity by hydroxylamine method. MDA level was raised while SOD activity was impaired in myocardial tissues from septic rats (Fig. 1H, I).

Bax and Bcl-2 mRNA expression in cardiomyocytes from septic rats were measured, as reflected by the fact that Bax was increased while Bcl-2 was decreased (Fig. 1J, K).

### miR-124-3p is lowly expressed while SP1, HDAC4, and HIF-1α are highly expressed in myocardial tissues of septic rats

**Fig. 2:**
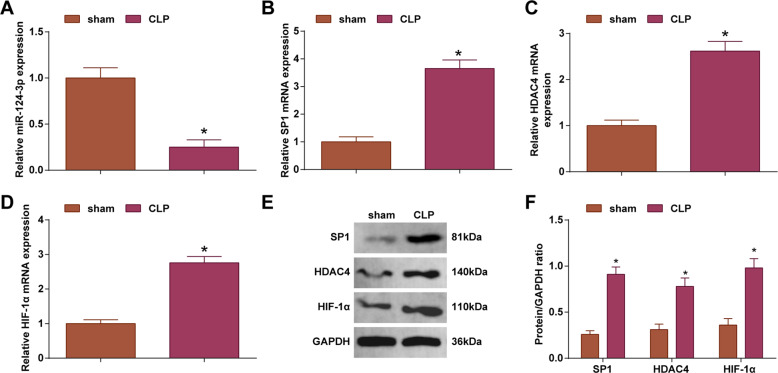
miR-124-3p is lowly expressed while SP1, HDAC4, and HIF-1α are highly expressed in myocardial tissues of septic rats. **A** RT-qPCR detected miR-124-3p expression in rat myocardial tissues; **B** RT-qPCR detected SP1 mRNA expression in rat myocardial tissues; **C** RT-qPCR detected HDAC4 mRNA expression in rat myocardial tissues; **D** RT-qPCR detected HIF-1α mRNA expression in rat myocardial tissues; **E** and **F** Western blot detected SP1, HDAC4, and HIF-1α protein expression in rat myocardial tissues; *n* = 6; the measurement data were expressed as mean ± standard deviation, *P < 0.05 compared with the sham group.

miR-124-3p stands in the conversion of metabolic substrates in heart disease [23]. HIF-1α is a key transcriptional activator induced under hypoxic conditions that is involved in hypoxic myocardial damage induced by endotoxin [24]. SP1 can target and regulate HIF-1 in myocardial injury [25]. HDAC4 can play a certain regulatory role in myocardial injury by reducing the production of reactive oxygen species [26] and HDAC4 can enhance the anti-activation effect of HIF-13 [27]. When evaluating the role of miR-124-3p/SP1/HDAC4/HIF-1α axis in myocardial injury in septic rats, miR-124-3p, SP1, HDAC4, and HIF-1α expression levels were detected by RT-qPCR and Western blot in myocardial tissues. The findings manifested downregulated miR-124-3p expression and upregulated SP1, HDAC4, and HIF-1 expression (Fig. 2A–F), indicating the involvement of miR-124-3p/SP1/HDAC4/HIF-1α axis in myocardial injury in septic rats.

### Upregulation of miR-124-3p ameliorates inflammation, oxidative stress, and apoptosis of LPS-treated cardiomyocytes

**Fig. 3:**
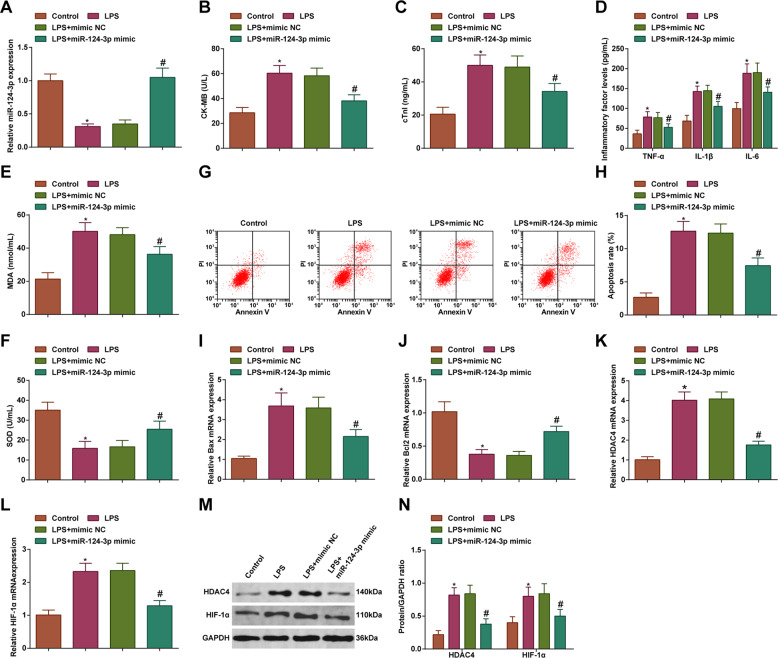
Upregulation of miR-124-3p ameliorates inflammation, oxidative stress, and apoptosis of LPS-treated cardiomyocytes. **A** RT-qPCR detected miR-124-3p expression level in cardiomyocytes; **B** ELISA detected CK-MB level in cardiomyocytes; **C** ELISA detected cTn-I level in cardiomyocytes; **D** ELISA detected TNF-α, IL-1β, and IL-6 levels in cardiomyocytes; **E** MDA level in cardiomyocytes; **F** SOD activity in cardiomyocytes; **G** and **H** Flow cytometry detected apoptosis of cardiomyocytes; **I** RT-qPCR detected Bax mRNA expression level in cardiomyocytes; **J** RT-qPCR detected Bcl-2 mRNA expression level in cardiomyocytes; **K** RT-qPCR detected HDAC4 mRNA expression level in cardiomyocytes; **L** RT-qPCR detected HIF-1α mRNA expression level in cardiomyocytes; **M** and **N** Western blot detected HDAC4 and HIF-1α protein expression in cardiomyocytes; the measurement data were expressed as mean ± standard deviation, repetition = 3, *P < 0.05 compared with the control group; ^#^*P* < 0.05 compared with the LPS + mimic NC group.

To figure out the impact of miR-124-3p/SP1/HDAC4/HIF-1α axis on cardiomyocytes in sepsis, in vitro experiments were conducted with cardiomyocytes treated with LPS. miR-103 has already been evidenced to downregulate in heart failure, suggesting its regulatory role in myocardial injury. For clarification of the mechanism of miR-124-3p in septic myocardial injury, miR-124-3p upregulation assay was performed. Experimental results indicated that LPS increased CK-MB, cTnI, TNF-α, IL-1β, IL-6, MDA, and Bax mRNA expression, suppressed SOD activity and Bcl-2 mRNA expression, and induced apoptosis of H9C2 cardiomyocytes. However, in response to miR-124-3p overexpression, LPS-induced damages to cardiomyocytes would be ameliorated (Fig. 3A–J). HDAC4 and HIF-1α expression levels were increased by LPS treatment while decreased by miR-124-3p upregulation (Fig. 3K–N). It was clear that overexpressing miR-124-3p reversed the myocardial injury induced by LPS.

### Silencing SP1 alleviates LPS-induced damages to cardiomyocytes

**Fig. 4:**
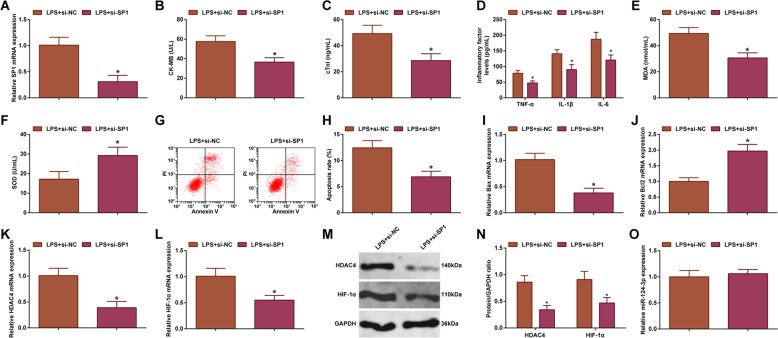
Silencing SP1 alleviates LPS-induced cardiomyocyte damage. **A** RT-qPCR detected SP1 mRNA expression in cardiomyocytes; **B** ELISA detected CK-MB level in cardiomyocytes; **C** ELISA detected cTn-I level in cardiomyocytes; **D** ELISA detected TNF-α, IL-1β, and IL-6 levels in cardiomyocytes; **E** MDA level in cardiomyocytes; **F** SOD activity in cardiomyocytes; **G** and **H** Flow cytometry detected apoptosis of cardiomyocytes; **I** RT-qPCR detected Bax mRNA expression in cardiomyocytes; **J** RT-qPCR detected Bcl-2 mRNA expression in cardiomyocytes; **K** RT-qPCR detected HDAC4 mRNA expression in cardiomyocytes; **L** RT-qPCR detected HIF-1α mRNA expression in cardiomyocytes; **M**–**N** Western blot detected HDAC4 and HIF-1α protein expression in cardiomyocytes; **O** RT-qPCR detected miR-124-3p expression in cardiomyocytes; the measurement data were expressed as mean ± standard deviation, repetition = 3, *P < 0.05 compared with the LPS + si-NC group.

SP1 could regulate myocardial injury [28]. In addition, up-/down-regulating SP1 could elevate/reduce HDAC4 expression [29]. HDAC4 exerts a critical role in myocardial injury [30–32]. Moreover, knocking down SP1 could decrease HIF-1α expression level [28]. To navigate the mechanism of SP1 in sepsis, SP1 expression was silenced in LPS-treated H9C2 cardiomyocytes, and there showed relieved cardiomyocyte damage and inflammation, suppressed oxidative stress and apoptosis, and reduced HDAC4 and HIF-1α expression levels (Fig. 4A–N). Furthermore, miR-124-3p expression was tested in cardiomyocytes after silencing SP1, and the results presented that silencing SP1 did not affect the expression of miR-124-3p (Fig. 4O). It was concluded that silencing SP1 alleviated LPS-induced damages to cardiomyocytes.

### miR-124-3p targets SP1; HDAC4 interacts with SP1

**Fig. 5:**
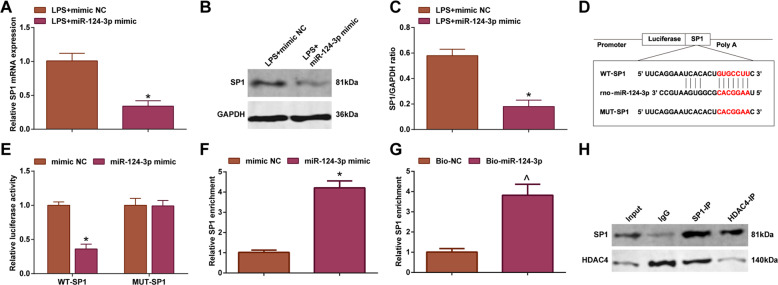
miR-124-3p targets SP1; HDAC4 interacts with SP1. **A** RT-qPCR detected SP1 mRNA expression after overexpression of miR-124-3p in LPS-induced cardiomyocytes; **B** and **C** Western blot detected SP1 protein expression level after overexpression of miR-124-3p; **D** Bioinformatics website predicted the binding site of miR-124-3p and SP1; **E** Dual luciferase reporter gene assay verified the regulatory relationship between miR-124-3p and SP1; **F** RIP assay verified the binding relationship between miR-124-3p and SP1; **G** RNA-pull down assay verified the binding relationship between miR-124-3p and SP1; **H** CO-IP assay detected the interaction between SP1 and HDAC4; the measurement data were expressed as mean ± standard deviation, repetition = 3, *P < 0.05 compared with the LPS + mimic NC group; ^*^P < 0.05 compared with the mimic NC group; ^*P* < 0.05 compared with the Bio-NC group.

Next, the potential connection between SP1 and miR-124-3p was explored. It was detected that treatment with LPS + miR-124-3p mimic decreased SP1 mRNA and protein expression (Fig. 5A–C). After that, the targeting relationship between miR-124-3p and SP1 was predicted in the bioinformatics software http://www.targetscan.org/vert_72/ (Fig. 5D). Moreover, dual luciferase reporter gene assay validated that miR-124-3p mimic destroyed the luciferase activity of Wt-SP1 (Fig. 5E). Further detected by RIP assay, a specific binding region was explored between SP1 and miR-124-3p (Fig. 5F). RNA-pull down assay presented that miR-124-3p enrichment was increased by Bio-SP1-WT (Fig. 5G). All of the results proved the targeting relation SP1 and miR-124-3p.

Next, CO-IP assay was performed to explore the interaction between HDAC4 and SP1. It was mirrored that SP1 and HDAC4 were co-precipitated in H9C2 cardiomyocytes by anti-SP1 antibody. Co-immunoprecipitation with anti-HDAC4 antibody further confirmed this interaction, indicating that HDAC4 bound and interacted with SP1 (Fig. 5H).

### SP1 overexpression antagonizes miR-124-3p upregulation-induced improvements in LPS-induced cardiomyocyte damage

**Fig. 6:**
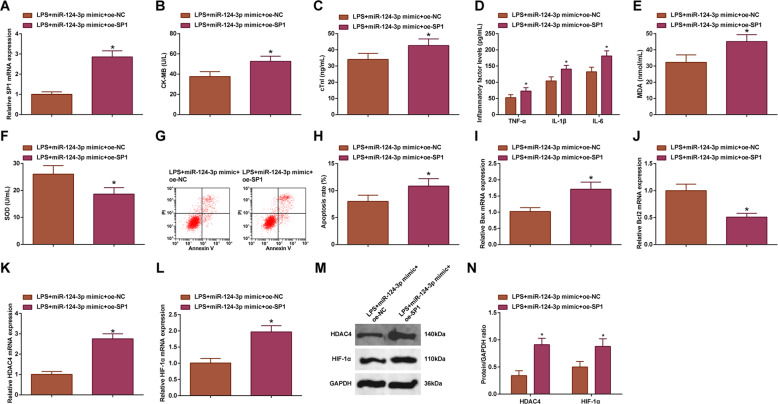
SP1 overexpression antagonizes miR-124-3p upregulation-induced improvements in LPS-induced cardiomyocyte damage. **A** RT-qPCR detected SP1 expression in cardiomyocytes; **B** ELISA detected CK-MB in cardiomyocytes; **C** ELISA detected cTn-I in cardiomyocytes; **D** ELISA detected TNF-α, IL-1β, and IL-6 levels in cardiomyocytes; **E** MDA level in cardiomyocytes; **F** SOD activity in cardiomyocytes; **G** and **H** Flow cytometry detected apoptosis of cardiomyocyte; **I** RT-qPCR detected Bax mRNA expression in cardiomyocytes; **J** RT-qPCR detected Bcl-2 mRNA expression in cardiomyocytes; **K** RT-qPCR detected HDAC4 mRNA expression in cardiomyocytes; **L** RT-qPCR detected HIF-1α mRNA expression in cardiomyocytes; **M** and **N** Western blot detected HDAC4 and HIF-1α protein expression in cardiomyocytes; the measurement data were expressed as mean ± standard deviation, repetition = 3, *P < 0.05 compared with the LPS + miR-124-3p mimic + oe-NC group.

The therapeutic effect of miR-124-3p upregulation on septic cardiomyocytes, as well as the targeting relationship between miR-124-3p and SP1 has been verified in our previous experiments, but whether SP1 was involved in miR-124-3p-mediated myocardial injury in sepsis was not clearly elucidated. To further confirm the combined performance of miR-124-3p and SP1 in sepsis, LPS-treated cardiomyocytes were co-transfected with miR-124-3p mimic and SP1 overexpression vector. Eventually, the results reflected that overexpression of SP1 would reverse miR-124-3p upregulation-induced effects on LPS-treated cardiomyocytes, and HDAC4 and HIF-1α expression levels (Fig. 6A–N). It could be summarized that spontaneous upregulation of miR-124-3p and SP1 aggravated cardiomyocyte damage in sepsis.

## Discussion

Sepsis initiates cardiac dysfunction, leading to high morbidity and mortality [11]. In the context of sepsis, severe myocardial injury and inflammation were manifested in animal models in this research. Through a series of assays, we testified the mechanism of sepsis-induced myocardial injury from miR-124-3p/SP1/HDAC4/HIF-1α axis. Exactly, miR-124-3p expression was restrained in myocardial tissues of septic rats and miR-124-3p upregulation in LPS-treated cardiomyocytes attenuated cellular inflammation, oxidative stress, and apoptosis. Actively, SP1 was overexpressed in sepsis and SP1 depletion relieved LPS-induced cardiomyocyte damage. In a word, miR-124-3p targeted SP1 to regulate HDAC4 and HIF-1α, thereby attenuating the pathology of sepsis-induced myocardial injury.

miR-124-3p maintains a low level in sepsis-related diseases that aggravates disease progression. As exampled by a report related to sepsis-induced lung injury, lowly expressed miR-124 has been measured and upregulating miR-124 relieves symptoms of lung injury through reducing inflammatory response and apoptosis, and promoting proliferation of lung tissue cells [8]. Further supported by a late experimental discovery, elevated miR-124 level is examined to suppress the levels of pro-inflammatory cytokine in sepsis [7]. More than that, miR-124 expression is reduced in patients with sepsis that is correlated with increased disease risk and inflammatory response [5]. More significantly, miR-124-3p is downregulated in myocardial ischemia-reperfusion injury and upregulating miR-124-3p could suppress apoptosis and inflammatory response of cardiomyocytes [33]. Consistently, miR-124-3p exerted the protective function in sepsis-induced myocardial injury.

SP1 was targeted by miR-124-3p in the present work, which shall be further validated. More importantly, overexpression of SP1 reversed the therapeutic effects of upregulated miR-124-3p on LPS-suffered cardiomyocytes. However, except for this research, there are various reports mentioning the concrete mechanism of SP1 in sepsis. SP1 is the positive regulator of ZFAS1 that worsens cardiac dysfunction induced by sepsis [11], suggesting the involvement of SP1 in sepsis. Functionally, silencing SP1 recovers cholinergic anti-inflammatory pathway activation and strains the neuro-inflammatory reaction and apoptosis induced by sevoflurane [34]. In addition to that, SP1 inhibition could cooperate with miR-375 to reduce oxidative stress, inflammation, neuronal apoptosis in Parkinson’s disease [35]. Specifically in myocardial injury, an inhibitor of SP1 (mithramycin) is disclosed to limit cardiomyocyte apoptosis [36] and si-SP1 transfection could attenuate myocardial hypoxia-induced endothelial cell apoptosis and oxidative stress [28]. Moreover, the positive rate of SP1 in myocardial ischemia-reperfusion injury (MI/RI) is enhanced and miR-374-mediated SP1 inhibition functions to impede cell apoptosis [37].

The interaction between HDAC4 and SP1 found in this work is supported by the fact that HDAC4 binds to SP1 [38]. HDAC4 has been implied as a potential treatment target for sepsis [39]. In sepsis-associated encephalopathy, HDAC4 is upregulated while HDAC4 inhibition suppresses Bax expression and neuronal apoptosis in vitro and in vivo [13]. In MI, overexpressed HDAC4 is the adverse inducer of exacerbated cardiac dysfunction and increased apoptosis [40]. However, depleting HDAC4 has been implicated to mechanistically repress the apoptosis of cardiomyocyte in diabetic cardiomyopathy [41]. Enhanced HDAC4 deteriorates the damage in MI/RI and reduces SOD-1 which would be functionally attenuated by HDAC inhibitors [14].

HDAC4 does interact with HIF-1α [42] which is in compliance with our study. Not only in this study, the upregulated HIF-1α has been tested upon LPS treatment [17]. Downregulation of HIF-1α in neonatal macrophages has been indicated to relate with the attenuation of sepsis-induced lung injury [43]. In sepsis-induced lung injury, silencing HIF-1α also presents its anti-inflammatory and anti-oxidant effects [44]. Actually, if suppressed in myocardial tissues of septic mice, HIF-1α could disturb the apoptosis of cardiomyocytes and the secretion of oxidative stress-related factors [18].

All in all, it was certified that miR-124-3p interacted with SP1 to suppress HDAC4, thereby ameliorating sepsis-induced myocardial injury, which may related to the knockdown of HIF-1α. This paper may widen our horizon to sepsis-related mechanism. Further studies are at wanting to deeply decode the miR-124-3p/SP1/HDAC4/HIF-1α axis in sepsis.

## Methods and materials

### Ethics statement

All experimental operations were carried out following the Guidelines for the Use of Laboratory Animal of the National Institutes of Health. The experimental protocol was approved by the Experimental Animal Ethics Committee of Nanfang Hospital, Southern Medical University.

### Animal treatment

Sprague Dawley rats (20 weeks; 220 ± 20 g) were available from the Experimental Animal Center of Guangzhou University of Chinese Medicine (Guangzhou, China). Fed for 7 days with (50 ± 3)% humidity, 12 h light/dark cycle, and sufficient food and water at (25 ± 1)°C, randomly selected rats (*n* = 10) were treated with CLP to establish a sepsis model. Fasted for 12 h, rats were anesthetized with 40 mg/kg pentobarbital sodium and fixed on the operating table. A middle incision was performed on the middle and lower abdomen to expose the cecum which was then ligated at 3/4 of the lower margin (avoid ligating the mesenteric vessels of the ileum and cecum). A small amount of content in cecum was squeezed out by perforating the distal and proximal ends with a 18-gauge needle. Afterward, the cecum was placed into the abdomen and the incision was sutured. After the operation, rats were given a subcutaneous injection of normal saline (1 mL) preheated at 37 °C, and a subcutaneous injection of tramadol hydrochloride as an analgesic at 10 mg/kg (once every 12 h within 24 h). Rats were recovered on a warm blanket and then kept in a separate cage with enough food and water supply. Except for cecum ligation, the other procedures for sham-operated rats were the same as the modeled rats [45, 46]. In addition, the rats were divided into two groups (*n* = 10): the sham group and the CLP group. The general condition of the rats was observed twice a day after CLP, and the rats were continuously monitored for 7 days, and the survival time was recorded.

### Left ventricular systolic pressure (LVSP) and left ventricular end diastolic pressure (LVEDP) detection

At 24 h post surgery, LVSP and LVEDP were monitored by a small animal ultrasound imaging system PanoViewb1500 (Nuohoi Life Sciences Co., Ltd., Shanghai, China) [47].

### Hematoxylin-eosin (HE) staining

Followed by LVSP and LVEDP detection, rats were euthanized to harvest myocardial tissues which were fixed in 4% formaldehyde, paraffin-embedded, and prepared into 4 μm sections. The baked tissues were sequentially immersed in xylene I, xylene II, absolute ethanol I, absolute ethanol II, as well as alcohol (95, 80, and 70%). Subsequently, the sections were stained with hematoxylin, rinsed in running water, immersed in 1% hydrochloric acid ethanol, and treated with 50, 70, and 80% alcohol. Next, the sections were immersed in eosin solution, processed by 95% ethanol, ethanol I, ethanol II, xylene I, and II, sealed with neutral glue, and inspected with a microscope [48, 49].

### Cell culture

H9C2 cardiomyocytes (ATCC, Rockville, MD, USA) were cultured in Dulbecco’s modified Eagle’s medium plus 10% fetal bovine serum and 100 μg/mL penicillin and streptomycin. The culture medium was renewed every 2–3 days during cell subculture [50, 51].

### Cell transfection

Regarding to the known SP1 and miR-124-3p sequences in NCBI, the plasmids were generated by Sangon (Shanghai, China). H9C2 cardiomyocytes at passage 3 were trypsinized and cultivated in 24-well plates at 2 × 10^5^ cells/well. Cardiomyocytes were transfected with si-SP1, miR-124-3p mimic, miR-124-3p mimic, and SP1 overexpression vector, as well as their corresponding negative control (NC). At 48 h post transfection, cardiomyocytes were processed with 10 μL/mL LPS or phosphate-buffered saline (PBS) for 24 h. Lipofectamine 2000 (Invitrogen, CA, USA) was utilized in cardiomyocyte transfection [52–54].

### Reverse transcription quantitative polymerase chain reaction (RT-qPCR)

Trizol (Life Technologies, Rockville, MD, USA) was employed to extract total RNA from tissues and cells. The RNA was reverse-transcribed into cDNA via a reverse transcription kit (Thermo, MA, USA). Gene expression was calculated with SYBR Green method on the ABI7500 fluorescent quantitative PCR instrument. U6 and glyceraldehyde-3-phosphate dehydrogenase (GAPDH) were considered as the internal controls. The data was analyzed by 2^-ΔΔCt^ method [28].

### Western blot assay

Myocardial tissues were homogenized with radio-immunoprecipitation assay (RIPA) lysis buffer on ice bath. H9C2 cardiomyocytes were lysed in RIPA lysis buffer containing protease inhibitors and centrifuged at 12,000 r/min. Protein concentration was measured by a bicinchoninic acid (BCA) kit (Junxin, Suzhou, China). Separated by 10% sodium dodecyl sulfate-polyacrylamide gel electrophoresis, the protein was mixed with the loading buffer, boiled at 100 °C, centrifuged in an ice bath, and electro-blotted onto a nitrocellulose membrane. The protein membrane sealed in 5% skim milk powder was reacted with the primary antibodies SP1 (1:500, Invitrogen), HDAC4 (1:100, Cell Signaling Technology, USA), HIF-1α (1:1000, Cell Signaling Technology, USA) and GAPDH (1:1000, Cell Signaling Technology). Then, the membrane was probed with horseradish peroxidase-conjugated secondary antibody (1:2000, Santa Cruz Biotechnology, Dallas, USA), developed by enhanced chemiluminescence and observed by Image Quant LAS 4000 C (General Electric, MA, USA) [18, 55].

### Enzyme-linked immunosorbent assay (ELISA)

Blood samples were collected from rat eyeballs and centrifuged at 3000 g/min to obtain the serum. H9C2 cardiomyocytes after transfection were centrifuged at 1000 r/min to collect the supernatant. Tumor necrosis factor-α (TNF-α), interleukin (IL)-1β and IL-6 levels, as well as Creatine kinase MB (CK-MB) and cardiac troponin I (cTnI) were examined by ELISA kit (Solarbio, Beijing, China) [47, 55].

### Detection of oxidative stress-related indices

Myocardial tissues were homogenized with normal saline at 1:9 and centrifuged at 3000 r/min to collect the supernatant. The protein was quantified by the BCA kit (Junxin). H9C2 cardiomyocytes were homogenized in 500 μL PBS via an ultrasonic cell disrupter and centrifuged at 1200 r/min to harvest the supernatant. Malondialdehyde (MDA) content and superoxide dismutase (SOD) activity in myocardial tissues and H9C2 cardiomyocytes were measured by the kit (Jiancheng Bioengineering Institute, Nanjing, China) [19, 21].

### Flow cytometry

Cell apoptosis was measured by flow cytometry. Cells were stained with Annexin V/PI Double Staining Kit (BD Biosciences, MA, USA) and detected on the FSCAN flow cytometer (BD Biosciences) [56].

### Dual luciferase reporter gene assay

TargetScan (http://www.targetscan.org/vert_72/) was utilized to predict the binding sites of SP1 and miR-124-3p, while dual luciferase reporter gene assay to verify the targeting relationship between them. Wild type (Wt)-SP1 and mutant (Mut)-SP1 were formed by pMIR-Report luciferase vector (Ambion, TX, USA). Next, H9C2 cardiomyocytes were co-transfected with Wt-SP1 or Mut-SP1 and miR-124-3p mimic or mimic NC via Lipofectamine 2000 (Invitrogen). H9C2 cardiomyocytes were collected to determine luciferase activity in a dual luciferase reporter gene detection system (Promega, Madison, WI, USA) [52].

### RNA immunoprecipitation (RIP) assay

EZMagna RIP kit (Millipore, MA, USA) was applied in RIP assay. H9C2 cardiomyocytes were lysed in RIP lysis buffer, reacted with magnetic beads bound to specific antibodies or control immunoglobulin G (Millipore), and incubated with proteinase K. Finally, the purified RNA was analyzed by RT-qPCR [57].

### RNA-pull down assay

Biotin-labeled Wt-SP1 (50 nM) and Mut-SP1 (50 nM) were transfected into cells, respectively. Then, lysed by a specific cell lysis buffer (Ambion), 50 mL cell lysate was packaged while others were reacted with M-280 streptavidin-coated magnetic beads (Dynabeads® M-280 Streptavidin, Search Biotech Co., Ltd., Beijing, China) which had been coated with RNase free and yeast tRNA (Sigma, St. Louis, MO, USA). The antagonistic miR-124-3p probe was applied as the NC. Total RNA was extracted by Trizol, and SP1 expression was evaluated by RT-qPCR [58].

### Co-immunoprecipitation (CO-IP) assay

CO-IP assay was performed with Dynabeads (Life Technologies, Germany). Cells were centrifuged at 12,000 r/min and acted with anti-HDAC4 and anti-SP1 antibodies overnight. Then, cells were cultivated with protein A/G agarose beads (100 μL), rinsed with RIPA buffer, and then subjected to Western blot assay. IgG served as the NC [59].

### Statistical analysis

All data were processed by SPSS 21.0 statistical software (IBM, NY, USA). Measurement data were expressed as mean ± standard deviation. Independent sample *t*-test was suitable for comparisons between two groups while one-way analysis of variance, along with Tukey’s post-hoc test for those among multiple groups. Comparisons at different time points were analyzed by repeated measures of analysis of variance and Bonferroni. *P* < 0.05 meant statistical significance.

## Supplementary information

aj-checklist

acciscovery-author-contribution-form

## Data Availability

The original contributions presented in the study are included in the article/Supplementary Material, further inquiries can be directed to the corresponding author.
